# Understanding household-level risk factors for zero dose immunization in 82 low- and middle-income countries

**DOI:** 10.1371/journal.pone.0287459

**Published:** 2023-12-07

**Authors:** Brooke Amara Farrenkopf, Xiaobin Zhou, Anita Shet, Folake Olayinka, Kelly Carr, Bryan Patenaude, Onyema Greg Chido-Amajuoyi, Chizoba Wonodi

**Affiliations:** 1 International Vaccine Access Center, Johns Hopkins Bloomberg School of Public Health, Baltimore, Maryland, United States of America; 2 United States Department of International Development, Immunization Team, District of Columbia, Washington, DC, United States of America; 3 Department of Epidemiology, The University of Texas MD Anderson Cancer Center, Houston, Texas, United States of America; Universitas Syiah Kuala, INDONESIA

## Abstract

**Introduction:**

In 2021, an estimated 18 million children did not receive a single dose of routine vaccinations and constitute the population known as zero dose children. There is growing momentum and investment in reaching zero dose children and addressing the gross inequity in the reach of immunization services. To effectively do so, there is an urgent need to characterize more deeply the population of zero dose children and the barriers they face in accessing routine immunization services.

**Methods:**

We utilized the most recent DHS and MICS data spanning 2011 to 2020 from low, lower-middle, and upper-middle income countries. Zero dose status was defined as children aged 12–23 months who had not received any doses of BCG, DTP-containing, polio, and measles-containing vaccines. We estimated the prevalence of zero-dose children in the entire study sample, by country income level, and by region, and characterized the zero dose population by household-level factors. Multivariate logistic regressions were used to determine the household-level sociodemographic and health care access factors associated with zero dose immunization status. To pool multicountry data, we adjusted the original survey weights according to the country’s population of children 12–23 months of age. To contextualize our findings, we utilized United Nations Population Division birth cohort data to estimate the study population as a proportion of the global and country income group populations.

**Results:**

We included a total of 82 countries in our univariate analyses and 68 countries in our multivariate model. Overall, 7.5% of the study population were zero dose children. More than half (51.9%) of this population was concentrated in African countries. Zero dose children were predominantly situated in rural areas (75.8%) and in households in the lowest two wealth quintiles (62.7%) and were born to mothers who completed fewer than four antenatal care (ANC) visits (66.5%) and had home births (58.5%). Yet, surprisingly, a considerable proportion of zero dose children’s mothers did receive appropriate care during pregnancy (33.5% of zero dose children have mothers who received at least 4 ANC visits). When controlled for other factors, children had three times the odds (OR = 3.00, 95% CI: 2.72, 3.30) of being zero dose if their mother had not received any tetanus injections, 2.46 times the odds (95% CI: 2.21, 2.74) of being zero dose if their mother had not received any ANC visits, and had nearly twice the odds (OR = 1.87, 95% CI: 1.70, 2.05) of being zero dose if their mother had a home delivery, compared to children of mothers who received at least 2 tetanus injections, received at least 4 ANC visits, and had a facility delivery, respectively.

**Discussion:**

A lack of access to maternal health care was a strong risk factor of zero dose status and highlights important opportunities to improve the quality and integration of maternal and child health programs. Additionally, because a substantial proportion of zero dose children and their mothers do receive appropriate care, approaches to reach zero dose children should incorporate mitigating missed opportunities for vaccination.

## Introduction

Routine immunizations are consistently regarded as one of the best investments in public health, and immunizations prevent an estimated 2 to 3 million deaths each year [1–3]. Over the past several decades, investments in immunization programs have led to extraordinary improvements in immunization access and utilization, and more children are reached by immunization services than many other preventative health services [4]. Routine immunization services are often one of a child’s first connections with the health system, so in addition to providing direct protection, immunization programs serve a unique opportunity to expand the reach and strength of the health system [4].

For nearly a decade between 2011 and 2019, coverage of the third dose of diphtheria-tetanus-pertussis (DTP) vaccination has been sustained around 85% (range: 84% to 86%). Yet, with the COVID-19 pandemic causing disruptions to health and routine immunization services, this global DTP3 coverage dropped from 86% in 2019 to 83% in 2020 and 81% in 2021 [5]. Therefore, in 2021, an estimated 25 million children were un- or under-vaccinated, an increase of 6 million children since 2019 [5–7]. Of those, an estimated 18 million children have not yet been reached with any immunization services in 2021 and constitute “zero dose” children [5,6]. The number of zero dose children in Gavi-supported countries had decreased by 1.7 million children over the previous four years, yet there were 5 million more zero dose children globally in 2021 than 2019 [5,8]. Immunization programs, particularly vaccination campaigns, have been successful at reaching children multiple times and have worked to increase the number of fully immunized children, yet there are currently more never-immunized than under-immunized children (defining zero dose children 12–23 months as having not received their first dose of DTP-containing vaccine and under-immunized children as having received at least the first but not the third dose of DTP-containing vaccine) [5,6]. This paradoxically highlights both the strength of routine immunization programs to provide continuous services, as well as the weakness of these programs to expand their reach to vulnerable populations over the past decade [5]. Reaching zero dose children is critical, as the same factors that contribute to them being unvaccinated will likely make them more vulnerable to vaccine-preventable diseases and hinder their ability to seek quality care if they become ill [9,10].

Currently, there is a concerted effort to address the gross inequity in immunization services and to reach zero dose children. This has been reflected in World Health Organization’s Immunization Agenda 2030 and Gavi, the Vaccine Alliance’s strategic plan for 2021 to 2025, suggesting that the next decade of immunization work will be largely focused on this area [11,12]. It is estimated that nearly half of zero dose children live in vulnerable contexts, including urban poor communities, remote rural areas, and conflict-affected settings [10]. Current approaches to reach children with vaccinations have proven to be insufficient in reaching entire communities and their children. Beyond identifying vulnerable contexts where zero dose children live, it is important to understand the risk factors and barriers they face in accessing care; this knowledge is essential to designing and implementing new approaches to engage vulnerable children in immunization systems and, more broadly, in the health system.

Several analyses have assessed factors related to under-immunization, but few analyses have looked specifically at zero dose immunization; if they did, they focused on specific contexts and were not multi-country or multi-regional analyses [13–16]. Household-level factors associated with poor immunization coverage include lower socioeconomic status, lower maternal education level, living in rural areas, and larger family size [13–22]. Children of mothers with low access to maternal care services, including tetanus toxoid immunization, antenatal care visits, and facility-based delivery, are more likely to be un- and under-immunized than fully immunized [14,16,20–22]. In some contexts, girls are more likely to be un- and under-immunized than boys; this finding was concentrated mainly in Southeast Asian countries [13,16,20]. Many studies have also looked at demand-related reasons for un- and under-vaccination, and common reasons include the following: being unaware of vaccine needs, concerns over vaccine safety, difficulty in traveling to the health facility, and caregiver not having time to take the child to the facility [13,21,23–25]. Demand-related factors varied considerably by context [13,21]. For the effort and investment in this area to lead to significant improvements and progress, it is central that we better understand the profile of zero dose children and the unique barriers that prevent them from successfully reaching immunization services. These barriers may differ substantially from those faced by under-immunized children who, although have sub-optimal engagement with the health system, have shown an ability to overcome access barriers to successfully receive immunization services. In addition, data suggest that children who receive at least one vaccine are likely to receive future vaccines, so understanding the truly never-immunized children is of great importance [6,26].

No recent analyses to our knowledge have quantified drivers of zero dose status at the global or multi-region level. With renewed attention to address immunization inequity by global organizations, it is essential to understand the population of zero dose children across countries, regions, and income levels, and avoid relying heavily on country-level analyses that may be influenced by specific contexts. Our study aims to build upon previously published literature and provides a recent analysis on risk factors of non-vaccination at the global level. A preceding analysis had similar approaches and utilized data from 1998 to 2008 to characterize unvaccinated children; this study was helpful in framing our work, but with renewed concentration on zero dose status, understanding the current burden and drivers of zero dose children is critical [17,20]. With the establishment of Gavi, the Vaccine Alliance in 1999 and concentrated investment and political support during the Decade of Vaccines beginning in 2010, the immunization landscape today is drastically different than it was two decades ago, and new analyses are needed to characterize the current landscape [27–30]. Additionally, recent work has helped to quantify zero dose status and describe patterns of under-immunization but has not quantified or ranked household demographic and health access factors as independent predictors of zero dose status [22,26,31]. With such investment and effort in this area, there is an urgent need to describe the population of zero dose children and understand the household-level drivers of zero dose status, so that programmatic levers can be applied to reach them.

The objective of our paper is to quantitatively and qualitatively describe the profile of zero dose children in low- and middle-income countries (LMICs) using recent, publicly available data. We propose a conceptual framework to describe the multilevel barriers specific to zero dose children based on the extant literature. We used this framework to guide our variable selection. We utilized descriptive and multivariate statistical analyses to identify household-level drivers of zero dose status globally and by country income level. We have developed this framework and conducted this analysis with child health and immunization managers in mind, so that their programs and efforts can address the barriers specific to zero dose children.

## Materials and methods

### Data sources

Our analysis utilized publicly available data collected through the Demographic and Health Surveys (DHS) and Multiple Indicator Cluster Surveys (MICS). The DHS program is supported by the United States Agency for International Development (USAID), and the MICS program is supported by the United Nations Children’s Fund (UNICEF). DHS and MICS are nationally representative household surveys conducted approximately every five years in low, lower-middle, and upper-middle income countries to collect information on demographic factors and maternal and child health service coverage and status. Both surveys follow a complex, two-stage cluster sampling design, and results from the surveys are representative at the national, regional, and residence (urban-rural) levels. The data utilized in our analysis are collected by the DHS and MICS teams via in-person interviews with women between 15 and 49 years of age, and caregivers are interviewed about the health of their children up to 59 months and the immunization history of their children 12 to 35 months (earlier DHS and MICS collected immunization data of children 12 to 59 months) [32–34].

Countries’ income levels were categorized into low, lower-middle, and upper-middle income countries based on the estimated per capita gross national income of the year that the survey was conducted, per the World Bank Group’s classification [35].

We utilized birth cohort estimates from the 2010 to 2020 United Nations Population Division’s (UNDP) World Population Prospects to provide a comparison for the study population as a proportion of the global population and population by country income levels [36]. UNDP estimates the number of births in five-year periods, so we utilized the number of births from 2010 to 2020 to align with the DHS/MICS data. UNDP utilized 2018 gross national income (GNI) data from the World Bank Group to classify country income level. We calculated the proportion of the birth cohort globally and in each country income group included in our analysis by totaling the estimated number of births in the countries in our analysis, divided by the total number of births globally and in the income group.

### Inclusion and exclusion criteria

Low income, lower-middle income, and upper-middle income countries were included in our analysis if they had a DHS or MICS published between 2011 and 2020 with immunization coverage data available in the survey. When multiple versions were available, we utilized the most recent full DHS or MICS published for each country. We excluded surveys that did not have data available on all of the childhood immunizations included in our outcome measure.

### Outcome measure

The main outcome, zero dose status, is defined as children ages 12 to 23 months at the time of the survey who have not received a single dose of the following routine immunizations: Bacille Calmette-Guérin (BCG), DTP-containing vaccine, inactivated or oral polio vaccine, and measles-containing vaccine. Children who have received at least one dose of these vaccines are not considered zero dose. Data were collected on these four vaccinations in the DHS/MICS; data for other vaccinations (i.e., pneumococcal or rotavirus vaccinations) were not consistently collected in DHS/MICS. Vaccination information was collected via vaccine card presentation, record of participation in vaccination campaigns (in MICS only), or caregiver recall when a vaccine card or record was not available.

Including children 12 to 23 months of age is aligned with recent analyses on zero dose status and with annual WHO/UNICEF estimates of national immunization coverage that considers this age group [5,10,22,26]. Including the cohort of children 12–23 months of age at the time of the survey reflects the youngest complete annual cohort that has had a chance to receive the full infant immunization schedule and therefore reflects the system performance of routine immunizations in the first year of life. Defining zero dose status by a set of routine infant immunizations, rather than one select immunization, such as DTP/pentavalent vaccine, provides a conservative estimate of zero dose children and more comprehensively assesses the reach of the immunization system. It is appropriate to use at the global level, as it aligns with the WHO vaccine recommendations [5,37]. Globally, all children should have received one or more doses of each of these vaccines by 12 months of age, and data are available for each of these vaccines after the first year of life in all DHS/MICS. In some contexts, second year of life vaccination programs have yet to be uniformly scaled up, so there would be more gaps in data if we considered children older than 24 months [38]. We also met with internal and external partners working in global vaccine delivery to ensure that our definition could be complementary to ongoing and planned work. In addition, this ensures a large enough sample size for our analysis.

### Framework development

Prior to beginning our analysis, a literature review was conducted to guide the development of a theoretical framework, define zero dose immunization, and to select covariates to include in our model. The literature review included studies published between January 2010 and February 2021, and considered zero dose immunization as an outcome (definitions of zero dose immunization differed across studies).

We identified a list of documented risk factors and concepts related to zero dose vaccination and grouped these variables by domains of interest. We then mapped these variables in a directed acyclic graph to identify the factors and areas that are more proximal and distal to zero dose status. Lastly, we considered available data sources that researchers and implementers could use when applying this framework to their work to conclude our final set of variables for the framework.

### Covariate selection

Through our literature review, we identified multiple indicators in the DHS/MICS to include in our analysis, related to household demographics and maternal and child health. The household demographic variables and how they were assessed in our analysis are as follows: residence (rural/urban living), wealth quintile (ordinal, with richest quintile as reference level), number of children ever born (1, 2–4, 5 or more), sex of head of household (male/female), frequency of listening to radio (less than weekly, at least weekly, almost daily), and frequency of watching television (less than weekly, at least weekly, almost daily). Maternal demographic factors include maternal education level (none, primary, secondary or more), mother’s age group (adolescent (15–19 years)/adult (20–49 years)), and marital status (never married, currently married, formerly married). Maternal and child health factors include the following: location of delivery (home, facility, or other), number of antenatal care (ANC) visits (0, 1–3, 4 or more), and number of maternal tetanus injections (0, 1, 2 or more). Child demographic and health factors include the following: sex of child (female/male), illness with fever in the past two weeks (yes/no), illness with cough in the past two weeks (yes/no), illness with diarrhea in the past two weeks (yes/no), treatment for recent cough/fever at a health facility (no/yes), and treatment for recent diarrhea at a health facility (no/yes). In the regression model, we used dummy variables for these categorical variables, and the reference was considered the least risk-related group (i.e., for the maternal tetanus variable, at least 2 tetanus injections was the reference group), based on findings in the literature. The exceptions to this were the wealth quintile variable, which we analyzed as an ordinal variable. In addition, primary education was the reference group for the maternal education variable, due to very limited access of secondary education in many settings in our analysis, which hindered the precision of our estimate and would have made interpretation of estimates challenging if based off widespread availability of secondary education; our approach for analyzing maternal education is similar to that of other studies [17,39].

### Statistical analysis

Descriptive statistics were provided to quantify global and national prevalence estimates of zero dose children. In pooling multicounty data from DHS and MICS, the original survey weights were adjusted according to the country’s population of children of 12–23 months of age [40,41]. We conducted univariate analyses to compare the characteristics of zero dose children among different country income levels. Chi-square tests with Rao & Scott’s second-order correction were conducted to compare the difference in the proportions of characteristics in zero dose and non-zero dose children. In addition, we stratified the overall population and zero dose population by urban/rural living and by wealth quintile, as considerable literature suggests that poor urban living is associated with undervaccination [10,42,43]. For the multivariate analysis, multiple imputation by chained equations was used to impute observations missing on one of the covariates of interest [41]. Multivariate logistic regressions were conducted to determine the association between zero dose status and the covariates pre-specified at the global level and stratified by country income level. In addition to the main covariates stated above, we controlled for country-specific effects, WHO region-specific effects, and the effect of different survey (DHS and MICS) in the analysis. All statistical analyses were conducted using R 4.0.4 with ‘survey’ package to consider the complex survey design in the analysis. Results were considered statistically significant at the alpha = 0.05 level.

## Results

Eighty-two countries were included in our analysis, with 28 low-income countries (34.1% of countries), 36 lower-middle income countries (43.9%), and 18 upper-middle income countries (22.0%) included. Through comparing the population in the countries in our analysis to the global cohort population estimates published by UNDP, the 82 countries in our analysis include 67.0% of the global birth cohort; when stratified, the countries in our analysis include 87.6% of the birth cohort of low-income countries, 95.4% of lower-middle income countries, and 28.6% of upper-middle income countries. The median survey year was 2017, and 75% of the surveys were published in 2015 or later. The majority of surveys were DHS (N = 47), and the remainder were MICS (N = 35).

### Theoretical framework

**Fig 1:**
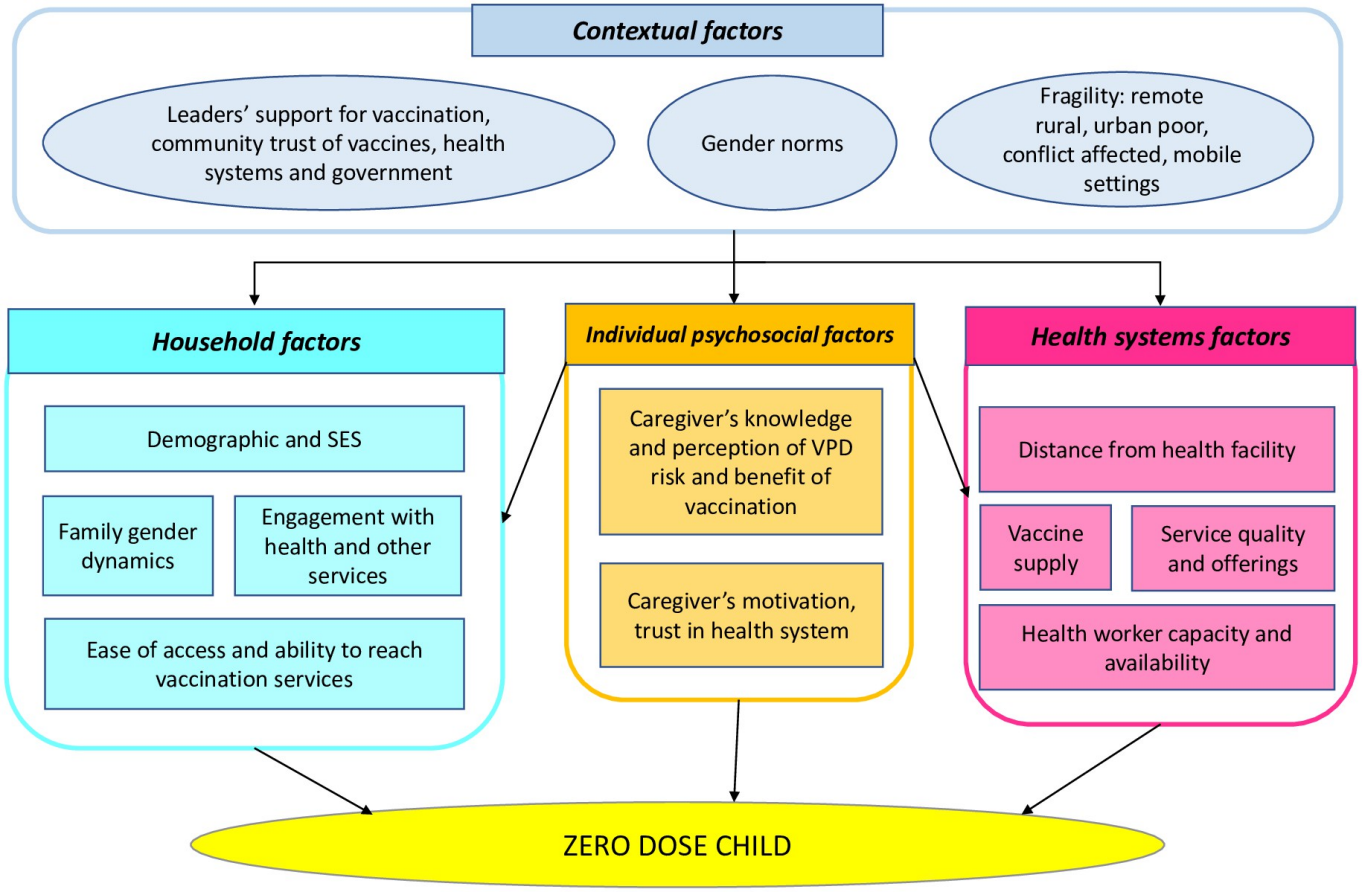


We developed a theoretical framework (Fig 1) to describe the multi-level correlates of zero dose status, by mapping the proximal and distal factors of zero dose status that are quantifiable and operational. The top level of the framework includes macro-level contextual factors related to community beliefs, political support, governance, gender norms, and location-related fragility [10,14,17,19,22]. The contextual factors affect the next set of more proximal factors: household, health system, and individual psychosocial factors. Household factors are related to sociodemographic status, engagement with services and resources (i.e., health and education services, access to media or internet), family-level gender dynamics (i.e., decision-making autonomy), and the ease of reaching vaccination services (i.e., ability to take work off or pay for transport to visit facility) [14,15,17–20]. Health systems factors consider distance to facility, programs and services offered at facilities, vaccine supply, quality of services, and health workforce capacity [16,19,20,39]. The set of psychosocial factors are the most proximal, as household demographics and health system factors affect individual psychosocial factors. Individual psychosocial factors include caregiver’s knowledge, attitudes, and perception toward vaccines and the risk of vaccine-preventable diseases, as well as trust in the health system and motivation to access health services [13,16,20,21]. Household-level factors like decision-making autonomy and ease of accessing services are likely to affect individual psychosocial factors, and past experiences in health facilities affect the trust in health system and motivation to seek services. Household factors are the focus of this analysis.

### Population prevalence of zero dose

**Fig 2:**
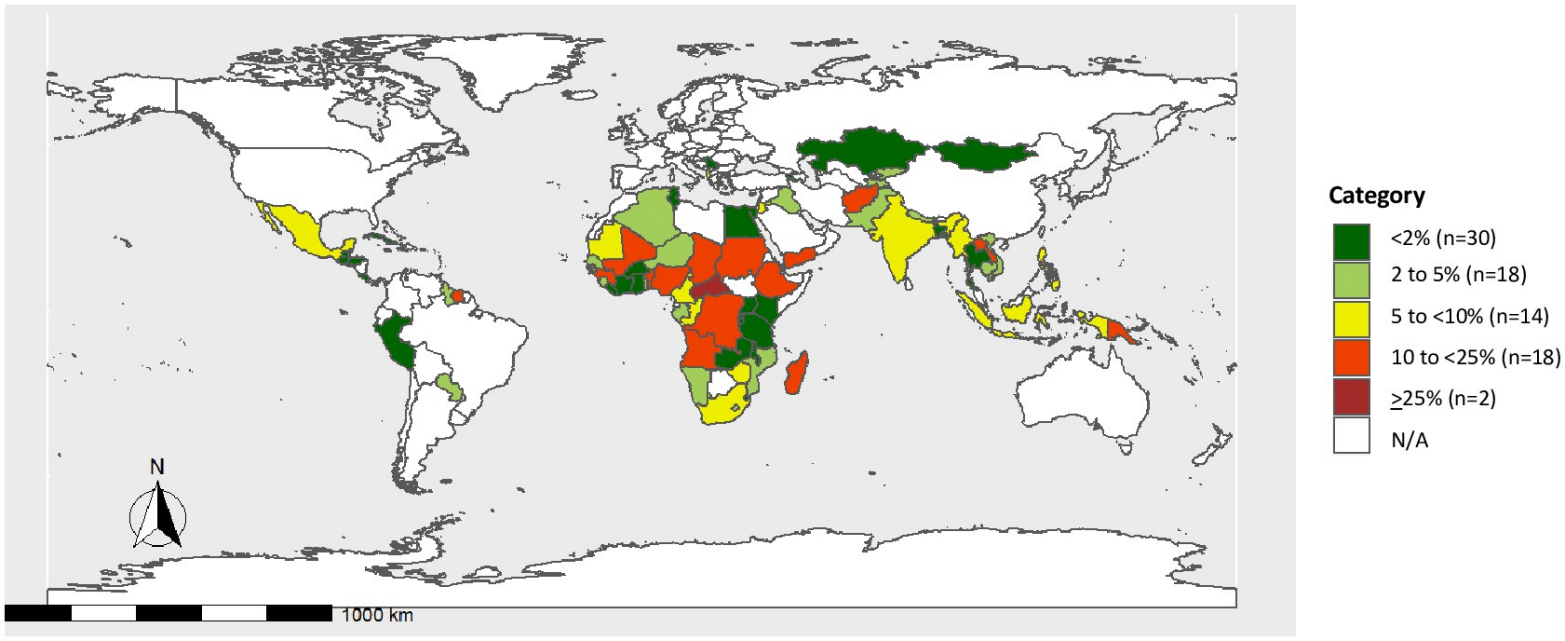


Within our study population, 7.5% of children (n = 14,697) were zero dose, having not received a single dose of Bacille Calmette-Guérin (BCG), DTP-containing vaccine, inactivated or oral polio vaccine, and measles-containing vaccine. Fig 2 shows the distribution of zero dose prevalence by country. In nearly a quarter of the countries in our analysis (n = 20), at least 10% of children 12 to 23 months are considered zero dose. The majority of countries with zero dose prevalence above 10% are located in the African region. (See S1 Appendix for national estimates of zero dose prevalence).

Table 1 shows the proportion of children considered zero dose by demographic and health system factors, globally and by country income group. In low-income countries, 1 in 10 children are zero dose (10.4%), followed by 7.0% in lower-middle income and 4.0% in upper-middle income countries. In the African region, 10.7% of children 12–23 months are zero dose, followed by 7.5% in the Western Pacific region, and 5.7% in both the South East Asia and Eastern Mediterranean regions. Nearly 9% (8.8%) of rural children are zero dose, which is higher than the global, non-stratified average of 7.5%. The highest proportion of zero dose children are in the poorest wealth quintile (12.4%), and the proportion of zero dose children decrease as wealth quintile increases; this trend is consistent in low-income and lower-middle income countries, but not upper-middle income countries where there is less variation by wealth quintile. Globally, 14.1% of children with mothers who haven’t received education are zero dose, which is nearly twice the global estimate of zero dose children. This finding is consistent in each of the country income levels, where we see an elevated proportion of zero dose status among mothers without education, compared to the zero dose prevalence in the general population. In low-income counties, 12.1% of children with adolescent mothers are zero dose. When looking at the proportion of zero dose children by access to maternal health services, we see elevated zero dose prevalence among children of mothers who have not received any tetanus injections (19.4%) or any antenatal care visits (24.0%), and 16.0% of children 12–23 months who were born at home are zero dose. We see similar zero dose prevalence estimates across maternal health indicators in low and lower-middle income countries, but not upper-middle income countries. In comparing the proportion of zero dose children by sex, the proportion of zero dose status in boys vs. girls is similar globally and across each country income group. Overall, 6.9% of children living in female-headed households are zero dose, which is slightly lower than the global average of 7.5%. Globally, 7.5% of children of mothers who are currently married and 8.2% of mothers who never married are zero dose, which is similar to the overall proportion of children zero dose (7.5%), with the exception of a relatively higher proportion of zero dose children with mothers who were never married than currently or formerly married in upper-middle income countries. Lastly, we see that there is a higher proportion of zero dose children among those whose mothers access TV and radio less than once a week (11.1% and 8.2%, respectively). (See S2 Appendix for the distribution of covariates in the entire population in our analysis (N = 194,829)).

**Table 1:** Proportion of children 12 to 23 months that are zero dose by demographic and health system characteristics, globally and by country income level.

	Global N = 194,829 (% of population)	Low-income countries N = 43,884 (% of population)	Lower-middle income countries N = 137,702 (% of population)	Upper-middle income countries N = 13,243 (% of population)
**Percent of population**	7.5	10.4	7.0	4.0
**WHO region**				
AFR	10.7	10.3	11.7	5.6
AMR	4.2	10.2	0.6	4.4
EMR	5.7	13.1	5.0	3.5
EUR	2.3	3.6	2.1	1.8
SEAR	5.7	0.0	5.8	0.6
WPR	7.5	0.0	7.5	1.0
**Rural**	8.8	11.9	7.9	3.2
**Urban**	5.3	6.4	5.2	4.4
**Sex of child**				
Male	7.5	10.2	7.0	3.9
Female	7.6	10.7	6.9	4.0
**Wealth Index in Quintiles**				
Richest	3.2	4.4	2.7	3.9
Richer	4.9	7.6	4.1	3.5
Middle	6.7	9.8	5.9	4.4
Poorer	8.9	11.8	8.7	3.3
Poorest	12.4	16.7	11.9	4.7
**Maternal Education**				
Secondary or higher	4.1	7.2	3.6	4.0
Primary	7.1	8.0	7.0	3.0
None	14.1	14.3	14.1	7.4
* Missing (%)*	10.4	19.6	7.0	3.8
**Number of children ever born**				
1	5.3	7.9	4.9	3.3
2–4	6.9	10.0	6.4	4.0
≥5	12.8	12.4	13.6	5.6
*Missing (%)*	11.1	20.7	7.5	4.1
**Adolescent age of mother (15–19 years)**	9.3	12.1	8.7	3.7
*Missing (%)*	11.0	19.6	8.0	3.8
**Marital status**				
Currently married	7.5	10.5	7.0	3.3
Formerly married	8.2	10.9	7.3	3.2
Never married	7.9	8.6	6.7	8.4
* Missing (%)*	10.1	18.3	8.2	3.5
**Female head of household**	6.9	10.7	5.7	4.1
**Access to maternal care**				
**Maternal Tetanus Injection**				
≥2 vaccinations	4.4	5.5	4.2	2.8
1 vaccination	5.6	7.0	5.3	3.9
0 vaccinations	19.4	18.1	20.8	4.6
* Missing (%)*	9.3	22.6	6.2	5.5
**Number of Antenatal Visit**				
≥4 Visits	3.9	5.0	3.8	3.0
1–3 Visits	6.5	7.8	5.9	6.6
0 Visit	24.0	28.2	22.9	11.2
* Missing (%)*	14.9	25.6	11.1	9.1
**Place of Delivery**				
Medical Facilities	4.2	6.3	3.8	3.5
Home	16.0	17.4	15.6	7.3
Other	9.0	10.9	8.1	2.4
* Missing (%)*	13.5	20.9	9.2	10.4
**Access to media**				
**Frequency of Listening to Radio**				
Almost daily	4.8	8.3	4.5	3.1
At least weekly	5.9	5.1	6.3	6.3
Less than once a week	8.2	12.5	7.1	3.9
* Missing (%)*	6.5	15.8	7.3	2.7
**Frequency of Watching TV**				
Almost daily	3.5	5.7	3.5	2.5
At least weekly	4.4	5.5	4.1	5.3
Less than once a week	11.1	11.5	11.2	5.8
* Missing (%)*	6.9	16.6	7.8	2.6
**Recent child illness**				
**Had diarrhea recently**	7.5	8.5	7.3	2.9
* Missing (%)*	1.5	0.5	0.6	14.3
**Had cough recently**	5.7	8.4	5.1	2.7
* Missing (%)*	1.4	0.4	0.5	14.1
**Had fever recently**	7.0	9.0	6.6	2.1
* Missing (%)*	4.9	0.4	5.4	14.1
**Treatment for child illness**				
**Among with diarrhea in the past two weeks**	(N = 38,411)	(N = 15,200)	(N = 20,457)	(N = 2,754)
Percentage who received treatment at medical facilities	5.7	5.7	5.9	2.0
* Missing (%)*	1.2	3.0	0.1	0.1
**Among with cough/fever in the past two weeks**	(N = 64,262)	(N = 22,814)	(N = 35,632)	(N = 5,816)
Percentage who received treatment at medical facilities	5.2	6.0	5.1	3.8
* Missing (%)*	7.1	9.7	5.9	4.3

Column totals may not add up due to rounding.

Table 2 shows the characteristics of the zero dose population. Globally, half (51.9%) of zero dose children are located in the African Region, followed by a quarter (28.6%) in the South East Asian Region. Three quarters (75.8%) live in rural areas, with 84.1% in rural areas in low-income countries, 74.5% in lower-middle income countries, and 28.1% in upper-middle income countries. For reference, 65.3% of global the population in the analysis lives in rural areas (S2 Appendix). Globally, 62.7% are in the bottom two wealth quintiles, with 37.5% in the poorest 20% of the population. Half of zero dose children (51.5%) have mothers without education; yet in upper-middle income countries, three quarters (75.1%) of mothers have completed at least secondary education. Globally, 30.5% of zero dose children live in families with at least four other children, and this is higher in low-income countries (38.8%). Additionally, the majority of zero dose children have mothers that are married (93.1%, globally). There was no substantial difference observed by sex of the child globally or by country income level.

**Table 2:** Distribution of demographic characteristics and potential risk factors among zero dose children, globally and by country income level.

	Global N = 14,697 (% of population)	Low-income countries N = 4,584 (% of population)	Lower-middle income countries N = 9,589 (% of population)	Upper-middle income countries N = 524 (% of population)
**WHO region**				
AFR	51.9*	89.5*	35.4*	24.7*
AMR	2.3*	1.4*	0.1*	51.6*
EMR	11.4*	8.7*	12.3*	19.4*
EUR	0.3*	0.4*	0.1*	3.3*
SEAR	28.6*	0.0*	43.8*	1.0*
WPR	5.4*	0.0*	8.3*	0.0*
**Rural living**	75.8*	84.1*	74.5*	28.1
**Sex of Child, female**	49.0	51.4	47.8	50.7
**Wealth Index in Quintiles**				
Richest	7.0*	6.9*	6.6*	13.8
Richer	12.3*	14.0*	11.3*	16.1
Middle	18.1*	19.3*	17.3*	22.9
Poorer	25.2*	23.9*	26.1*	19.6
Poorest	37.5*	35.9*	38.8*	27.7
**Maternal Education**				
Secondary or higher	26.6*	16.6*	28.7*	75.1*
Primary	21.9*	27.2*	19.8*	16.2*
None	51.5*	56.2*	51.5*	8.8*
*Missing (%)*	0.7	1.4	0.3	1.8
**Number of children ever born**				
1	19.1*	14.9*	20.8*	25.8
2–4	50.4*	46.3*	51.7*	60.6
≥5	30.5*	38.8*	27.5*	13.6
* Missing (%)*	0.7	1.5	0.3	2.1
**Adolescent age of mother (15–19 years)**	7.5*	9.5*	6.5*	7.8
* Missing (%)*	0.7	1.4	0.3	1.8
**Marital status**				
Currently married	93.5	88.8	96.8	79.7*
Formerly married	3.4	6.3	2.2	7.1*
Never married	3.1	4.8	1.5	13.2*
*Missing (%)*	0.4	0.6	0.2	2.0
**Female head of household**	15.0*	18.3	13.0*	24.9
**Access to maternal care**				
**Maternal Tetanus Injection**				
≥2 vaccinations	37.6*	33.8*	38.8*	43.8
1 vaccination	15.5*	18.9*	13.2*	41.8
0 vaccinations	46.9*	47.2*	48.0*	14.4
* Missing (%)*	18.0	27.2	12.5	37.0
**Number of Antenatal Visit**				
≥4 visits	33.5*	26.4*	34.4*	77.2*
1–3 visits	26.6*	35.0*	23.5*	17.7*
0 visits	39.9*	38.6*	42.2*	5.1*
* Missing (%)*	14.3	21.5	10.4	21.9
**Place of Delivery**				
Medical Facilities	40.7*	38.2*	39.5*	88.8*
Home	58.5*	60.7*	59.9*	11.1*
Other	0.8*	1.1*	0.7*	0.2*
* Missing (%)*	1.5	2.4	0.6	9.2
**Access to media**				
**Frequency of Listening to Radio**				
Almost daily	2.5*	2.7*	1.8*	15.3*
At least weekly	13.9*	13.0*	13.0*	40.5*
Less than weekly	83.6*	84.3*	85.2*	44.1*
* Missing (%)*	5.1	2.3	5.8	16.8
**Frequency of Watching TV**				
Almost daily	9.6*	1.7*	13.0*	21.7*
At least weekly	16.7*	8.8*	19.7*	37.7*
Less than weekly	73.7*	89.5*	67.3*	40.7*
* Missing (%)*	5.7	2.3	6.7	16.0
**Child illness**				
**Had diarrhea recently**	19.2	20.2*	19.0	11.9
* Missing (%)*	1.7	1.0	1.5	13.4
**Had cough recently**	19.2*	21.3*	18.3*	17.6
* Missing (%)*	1.5	0.9	0.5	11.6
**Had fever recently**	23.8*	23.5*	24.7	10.4*
* Missing (%)*	4.5	0.6	6.0	11.9
**Treatment for child illness**				
**Among with diarrhea in the past two weeks**	(N = 3,140)	(N = 1,456)	(N = 1,603)	(N = 81)
Percentage who received treatment at medical facilities	43.3*	29.4*	50.7*	32.5*
* Missing (%)*	0.5	1.0	0.0	1.2
**Among with cough/fever in the past two weeks**	(N = 4,624)	(N = 2,071)	(N = 2,371)	(N = 182)
Percentage who received treatment at medical facilities	46.6*	29.3*	54.6*	76.1*
*Missing (%)*	7.1	6.3	7.8	7.1

Column totals may not add up due to rounding.

* denotes statistically significant difference between zero dose and non-zero dose children, p<0.05.

Nearly half of the mothers of zero dose children did not receive any tetanus vaccines (46.9%), 39.9% did not receive any ANC visits, and 58.5% had home births. Yet, it is important to note that 40.7% of zero dose children were delivered in a facility, and a third (33.5%) have mothers who received at least four ANC visits, highlighting potential missed opportunities. This is more pronounced in upper-middle income countries, where the majority of zero dose children have mothers who delivered in facilities (88.8%) and had at least four ANC visits (77.2%). Overall, 7.5% of zero dose children have an adolescent mother (15–19 years), and this is higher in low income countries (9.5%). Globally, 15.0% of households of zero dose children are female-headed. Lastly, that majority of zero dose children are in households with limited access to and utilization of media, with 73.7% and 83.6% of zero dose children having mothers that watch TV or listen to the radio, respectively, less than weekly. In comparing across country income levels, zero dose children are in households with more frequent access to radio and TV in upper-middle income countries (59.3% have mothers who watch TV at least weekly or almost daily in upper-middle income countries), but in households with less access to TV in low-income countries (89.5% have mothers who watch TV less than once a week in low-income countries). Globally, approximately 1 in 5 zero dose children experienced illness in the past two weeks: 19.2% of zero dose children had diarrhea in the past two weeks, 19.2% had cough, and 23.8% had fever. In looking at children with recent illness, less than half of children with recent diarrhea or cough/fever received treatment at a medical facility (43.3% and 46.6%, respectively) at the global level. This was lower in low-income countries, where less than 1 in 3 children with recent illness received treatment at medical facilities for diarrhea (29.4%) and cough/fever (29.3%).

Table 3 examines the relationship between urban/rural living, household wealth, and zero dose status. The leftmost columns in Table 3 provide a description of household wealth and population distribution in urban and rural areas in the entire study population. This provides a reference for the finding that there are more zero dose children in rural areas globally and in low-income and lower-middle income countries. There is a substantially higher proportion of children in rural areas in low- and lower-middle income countries, so it is expected that this would also be reflected in the distribution of zero dose children in these areas (S2 Appendix). There are more zero dose children in urban areas (71.9%) in the upper-middle income countries included in our analysis.

**Table 3:** Distribution of full population and zero dose population by urban/rural living and wealth quintile.

	Full population		Zero dose population							
	Total population in analysis (regardless of vaccination status)		Total zero dose population in analysis		Zero dose population in each country income group					
	Globally (N = 194,829)		Globally (N = 14,697)		Low-income countries (N = 4,584)		Lower-middle income countries (N = 9,589)		Upper-middle income countries (N = 524)	
Wealth quintile	Urban	Rural	Urban	Rural	Urban	Rural	Urban	Rural	Urban	Rural
Richest	12.8	3.7	5.4	1.6	5.2	1.7	5.0	1.6	13.2	0.5
Richer	10.2	8.9	6.8	5.5	6.4	7.7	6.6	4.7	15.0	1.0
Middle	6.1	14.3	5.6	12.5	2.1	17.2	6.6	10.6	17.4	5.6
Poorer	3.5	17.7	3.9	21.3	1.2	22.8	4.8	21.3	12.2	7.4
Poorest	2.0	20.8	2.5	35.0	1.1	34.8	2.5	36.3	14.1	13.6
**Total**	**34.7**	**65.3**	**24.2**	**75.8**	**16.0**	**84.1**	**25.5**	**74.5**	**71.9**	**28.1**

The leftmost results column contains the full population in the analysis, regardless of vaccination status; the remainder of the columns contain the zero dose population. Column totals may not add up due to rounding.

Overall, about 35% of children in the sample live in urban areas, with more of the urban households in the top two wealth quintiles (23.0%) than in the bottom two (5.5%). In urban areas, the zero dose population is also distributed more toward higher wealth quintiles. However, this is more a function of the wealth distribution of the urban population than the likelihood of being zero dose among the richer quintiles, given that 12.8% of all children live in the richest urban households but only 5.4% of zero dose children live in the richest urban households. It is worth noting that the urban poor population is small–only 2%–so contributions to the zero dose burden from such households will be limited in size. With 2.5% of all zero dose children from urban poor homes, this constitutes a higher contribution that would be expected given the distribution of children from urban poor households in the general population. We also find that the zero dose population in rural areas is made up more of the lower wealth quintiles. Over half of zero dose children globally and in low-income and lower-middle income countries are in the poorest 40% of rural households. This differs in upper-middle income countries, where 45.6% of the zero dose children are in the wealthiest 60% of urban households.

### Profile of zero dose children

Zero dose children are predominantly concentrated in the poorest two wealth quintiles. Globally, zero dose children have mothers who have not received education and live in households with limited access to TV and radio. In addition, the mothers of zero dose children have limited access to maternal health services, including tetanus injections, antenatal care, and health facilities for delivery; this is seen at the global level and mainly in low and lower-middle income countries.

Zero dose children–and the mothers of zero dose children–in upper-middle income countries are qualitatively different from zero dose children in lower income countries. There is a substantial proportion of zero dose children in each wealth quintile in upper-middle income countries, compared to primarily the bottom two wealth quintiles in the lower income countries. While zero dose children have mothers who haven’t received education in low and lower-middle income countries, zero dose children in upper-middle income countries have mothers who have received secondary education. The mothers of zero dose children in upper-middle income countries have moderate to high use of maternal health services, are likely to deliver in health facilities, and have moderate access to TV and radio, compared to low access in lower income countries.

### Multivariate analysis of zero dose risk factors

Sixty-eight countries were included in our multivariate analysis. Fourteen countries were excluded (one low-income country, six lower-middle income countries, and seven upper-middle income countries) because they did not have data on one or more of the pre-specified covariates. S3 Appendix presents the differences between the countries included and excluded in this analysis. Zero dose prevalence was comparable in included vs. excluded upper middle income countries (3.8% vs. 4.1%, respectively), but zero dose prevalence was higher in included than excluded lower-middle income countries (7.2% vs. 4.6%, respectively; excluded lower-middle income countries accounted for 7.4% of the sample size in lower-middle income countries.) The access to media and access to maternal tetanus injections had the most missing data. (Because only one low-income country was excluded, accounting for 1.1% of the sample size in low-income countries, the differences in included vs. excluded low-income countries are not reported.) Table 4 presents the odds ratios and 95% confidence intervals of potential drivers of zero dose status. When controlled for the other covariates included in the model, the strongest risk factor for zero dose status at the global level was non-receipt of maternal tetanus injections. Children of mothers who did not receive any tetanus injections have three times the odds of being zero dose than children of mothers who received at least two tetanus injections (OR = 3.00, 95% CI: 2.72, 3.30), and similar and statistically significant findings were seen across the income levels. Comparatively, low receipt of tetanus injections (1 dose) was a weaker, but statistically significant, driver of zero dose status globally (OR = 1.22, 95% CI: 1.10, 1.36). Children delivered at home were nearly twice as likely to be zero dose compared with children delivered at facilities (OR = 1.87, 95% CI: 1.70, 2.05); similar and statistically significant results were seen across the country income levels. Children of mothers who did not receive any ANC visits were nearly 2.5 times as likely to be zero dose than children of mothers who received at least 4 ANC visits (OR = 2.46, 95% CI: 2.21, 2.74), and children of mothers with low access to ANC visits (1–3 ANC visits) were 33% more likely to be zero dose (OR = 1.33, 95% CI: 1.21, 1.46) than children of mothers with at least 4 ANC visits. Children who did not receive treatment for fever/cough were 30% (95% CI: 16%, 47%) more likely to be zero dose than children who did receive treatment; no access to diarrhea treatment was marginally associated with zero dose status (OR = 1.14, 95% CI: 1.00, 1.30). In low-income countries, the lack of treatment for fever/cough (OR = 1.35, 95% CI: 1.09, 1.67) and diarrhea (OR = 1.35; 95% CI: 1.04, 1.74), compared to treatment in a medical facility, were risk factors for zero dose status, but the strength and statistical significance of these associations varied across the other country income levels.

**Table 4:** Odds ratios and 95% confidence intervals of potential risk factors for zero dose status, by income level, globally and by country income level.

Potential driver of zero dose status	Global (N* = 167,802.9) OR (95 CI)	Low-income countries (N* = 53,794.94) OR (95 CI)	Lower-middle income countries (N* = 101,417.1) OR (95 CI)	Upper-middle income countries (N* = 1,2303.2) OR (95 CI)
**Access to maternal care**				
Maternal tetanus injection				
≥2 vaccinations	1.00	1.00	1.00	1.00
1 vaccination	**1.22 (1.10, 1.36)**	**1.03 (0.86, 1.24)**	**1.36 (1.20, 1.55)**	1.23 (0.73, 2.09)
0 vaccinations	**3.00 (2.72, 3.30)**	**2.34 (1.91, 2.86)**	**3.28 (2.94, 3.66)**	**2.93 (1.82, 4.73)**
Delivery in facility	1.00	1.00	1.00	1.00
Delivery at home	**1.87 (1.70, 2.05)**	**1.79 (1.50, 2.14)**	**1.91 (1.71, 2.13)**	**1.69 (1.04, 2.75)**
ANC visits				
≥4 visits	1.00	1.00	1.00	1.00
1–3 visits	**1.33 (1.21, 1.46)**	**1.35 (1.14, 1.60)**	**1.32 (1.18, 1.48)**	**1.78 (1.07, 2.95)**
0 visits	**2.46 (2.21, 2.74)**	**3.22 (2.61, 3.97)**	**2.27 (2.00, 2.58)**	**2.50 (1.16, 5.37)**
**Access to childcare**				
Treated child’s fever/cough at medical facility				
Yes	1.00	1.00	1.00	1.00
No	**1.30 (1.16, 1.47)**	**1.35 (1.09, 1.67)**	**1.29 (1.12, 1.49)**	1.12 (0.66, 1.92)
Treated child’s diarrhea at medical facility				
Yes	1.00	1.00	1.00	1.00
No	1.14 (1.00, 1.30)	**1.35 (1.04, 1.74)**	1.07 (0.91, 1.25)	1.41 (0.67, 3.00)
**Maternal education**				
None	**1.32 (1.20, 1.46)**	**1.31 (1.10, 1.56)**	**1.32 (1.17, 1.49)**	**1.59 (0.98, 2.56)**
Primary	1.00	1.00	1.00	1.00
Secondary or higher	**0.85 (0.76, 0.95)**	0.96 (0.78, 1.18)	**0.81 (0.71, 0.93)**	0.75 (0.44, 1.30)
**Access to media**				
Watch TV				
Almost daily	1.00	1.00	1.00	1.00
At least weekly	1.16 (0.99, 1.36)	1.76 (0.94, 3.29)	1.10 (0.92, 1.30)	**2.00 (1.13, 3.53)**
Less than weekly	**1.37 (1.20, 1.56)**	1.62 (0.90, 2.93)	**1.39 (1.20, 1.60)**	**2.71 (1.66, 4.43)**
Listen to radio				
Almost daily	1.00	1.00	1.00	1.00
At least weekly	1.08 (0.85, 1.36)	1.01 (0.68, 1.51)	1.07 (0.79, 1.45)	0.78 (0.34, 1.79)
Less than weekly	1.20 (0.97, 1.48)	1.24 (0.87, 1.76)	1.12 (0.84, 1.48)	1.36 (0.68, 2.71)
**Household demographics**				
Urban	1.00	1.00	1.00	1.00
Rural	0.91 (0.81, 1.02)	1.11 (0.88, 1.40)	**0.87 (0.76, 0.99)**	0.85 (0.53, 1.37)
Sex of child				
Male	1.00	1.00	1.00	1.00
Female	1.01 (0.94, 1.08)	1.08 (0.96, 1.22)	0.97 (0.89, 1.05)	1.38 (0.96, 1.99)
Wealth quintile				
Richest	1.00	1.00	1.00	1.00
Ordinal^a^	**1.09 (1.05, 1.13)**	**1.13 (1.05, 1.21)**	**1.06 (1.01, 1.11)**	1.15 (0.97, 1.36)
Birth order				
1 (first born)	1.00	1.00	1.00	1.00
2–4	1.00 (0.90, 1.10)	1.05 (0.84, 1.31)	0.96 (0.86, 1.08)	1.82 (0.93, 3.53)
≥5	1.04 (0.92, 1.18)	1.06 (0.83, 1.35)	1.04 (0.89, 1.21)	1.33 (0.61, 2.91)
Mother’s age				
Adult (≥20 years)	1.00	1.00	1.00	1.00
Adolescent (15–19 years)	**1.31 (1.13, 1.52)**	**1.37 (1.05, 1.78)**	**1.27 (1.06, 1.52)**	1.75 (0.84, 3.67)
Marital status				
Currently married	1.00	1.00	1.00	1.00
Formerly married	1.16 (0.91, 1.47)	1.19 (0.80, 1.75)	1.03 (0.81, 1.31)	1.12 (0.38, 3.26)
Never married	0.88 (0.71, 1.10)	0.94 (0.66, 1.35)	0.76 (0.56, 1.01)	1.85 (0.78, 4.38)
Sex of head of household				
Male	1.00	1.00	1.00	1.00
Female	0.93 (0.83, 1.03)	1.05 (0.86, 1.28)	0.87 (0.76, 0.99)	0.66 (0.33, 1.31)

Bolded results are significant at p<0.05.

*Weighted sample size.

^a^ Analyzed as a linear ordinal variable.

Globally, children of mothers who did not receive education were 32% (95% CI: 20%, 46%) more likely to be zero dose than children of mothers who completed only primary education. Similar results were seen across country income levels. At the global level, secondary education was protective against zero dose status (OR = 0.85, 95% CI: 0.76, 0.95), compared to only primary education. In addition, limited access to television was a risk factor for zero dose status at the global level, with children of mothers who watch TV less than weekly having 1.37 (95% CI 1.20, 1.56) times the odds of zero dose status than children of mothers who watch TV almost daily. The effect of TV access is strongest in upper-middle income countries, as children whose mothers watch TV at least weekly (less than daily) and less than weekly have 2.00 (95% CI: 1.13, 3.53) and 2.71 (95% CI: 1.66, 4.43) times the odds of being zero dose than children of mothers who watch TV almost daily. Access to radio was not a driver of zero dose status globally or in any country income level.

Although a larger proportion of zero dose children live in rural areas (Table 2), when controlled for other covariates in the model, urban/rural living was not a significant driver of zero dose status globally (OR = 0.91, 95% CI: 0.81, 1.02) and in low and upper-middle income countries; in lower-middle income countries, rural living was marginally associated with a lower likelihood of being zero dose (OR = 0.87, 95% CI: 0.76, 0.99). In addition, the sex of the child was not a statistically significant driver of zero dose status globally (OR = 1.01, 95% CI: 0.94, 1.08) or in any country income level. Children of adolescent mothers (15 to 19 years), compared to children with mothers at least 20 years of age, are 31% (95% CI: 13%, 52%) more likely to be zero dose at the global level, and similar trends are observed across country income levels (although not significant in upper-middle income countries). Poor household socioeconomic status is also a risk factor for zero dose status; when considering wealth quintile as a linear ordinal variable, there is an additional 9% (95%: CI 5%, 13%) greater odds of being zero dose as one moves to the next poorer wealth quintile at the global level. In low-income countries, this descent to poor wealth was associated with a 13% (95% CI: 5%, 21%) increased odds of being zero dose. The negative association of wealth with zero dose status was not statistically significant in upper-middle income countries. Overall, family size, marital status, and sex of head of household were not found to be drivers of zero dose status (although female head of household was marginally associated with lower odds of zero dose status in lower-middle income countries).

## Discussion

Our analysis aimed to understand the current population of zero dose children and their risk factors, to aid in identifying, adapting, and targeting interventions to strategically reach them. A lack of access to maternal health care was a strong risk factor of zero dose status and highlights important opportunities to improve the quality and integration of maternal and child health programs. Limited maternal education and adolescent age also affect zero-dose status, highlighting the need for gender-responsive approaches to reach zero-dose children and their families.

An interesting finding was identifying that a substantial proportion of zero dose children and their mothers are engaged with the health system, and this highlights an opportunity to improve existing health services and mitigate missed opportunities for immunization. This shows that the health system reaches a substantial proportion of zero dose children and their families, but the routine immunization systems or campaigns still fail to reach these children in these contexts. It begs three important questions: 1) How can we improve the quality and scope of these maternal and child health programs and integrate their services with immunization services?, 2) What motivation and access factors drive families to seek health services for these areas that could be transferable for preventative, repeat immunization services?, and 3) Understanding key drivers, what approaches can be used to increase uptake of maternal services such as ANC and maternal immunization and also reduce missed opportunities for increasing routine immunization? A major missed opportunity is seen when we look at the 40.7% of zero dose children who were born in facilities but did not receive the birth-dose BCG vaccination. In addition, a third of zero dose children had mothers who received four or more ANC visits. One of the secondary objectives of ANC is to provide mothers with information, resources, and a pathway to protect themselves and their infants against vaccine-preventable diseases. Optimizing this process–or better, understanding its shortcomings–could be critical to reaching zero dose children [44]. Although some zero dose children remain entirely excluded from the health system, discussed next, it’s important to understand that a large proportion of zero dose children and their families do have existing connections with the health system, and addressing these missed opportunities could have remarkable impact on lessening the number of zero dose children.

A perhaps more urgent challenge is the proportion of zero dose children that are entirely disengaged from the health system; this brings up questions about health system reach and responsiveness to some communities. A third of zero dose children had mothers who did not receive any ANC visits prior to birth, and nearly 60% of zero dose children were born at home. In the multivariate analysis, covariates related to a lack of access to maternal health services were among the strongest drivers of zero dose status when controlled for other factors, so it is crucial to improve these services, both to reach women with essential services and to mitigate the zero dose burden. When comparing no access to low access to maternal health services in the multivariate analysis, interestingly, low access to maternal health services was a substantially weaker driver of zero dose status (OR = 1.22 for low access to tetanus injections and OR = 1.33 for low access to ANC) in comparison to no access to maternal health services (OR = 3.00 for no access to tetanus injections and OR = 2.46 for no access to ANC), suggesting that there are qualitative differences in those who never access health services and are disengaged from the health sector, versus those who have some level of engagement with the health system. Our findings align with the literature identifying the correlates of non-vaccination, and further reinforces the need for interventions and activities to address the challenges underlying children unreached by immunization services [13–18,22,23]. In addition, though, we also focus on the zero dose children who have mothers who do access appropriate care, thus shifting the discussion toward quality of care and missed opportunities for vaccination.

When looking at the immunization status of children of mothers with poor access to health care (i.e., no ANC visits), we see that the majority of the children of mothers with poor access to care do end up receiving immunizations and are not zero dose. Understandably, some of the same barriers that mothers may face in accessing their own care are likely to also be present when accessing immunization services for their children. Yet, we see that most of the mothers with poor health access are able to overcome these barriers: 76.0% of children of mothers with 0 ANC visits and 80.6% of children of mothers without tetanus injections do successfully receive immunization services and are not zero dose. Although this analysis did not assess this relationship, perhaps the relatively high percentage of women who did not receive ANC or tetanus injections but were able to get their children vaccinated show the strength of community outreach of vaccination programs. This leads us to question why a relatively small proportion of this low-access group is unable to overcome these barriers, and why they might face unique challenges and experience additional vulnerability that make them unlike their peers in accessing immunization services for their children. This could also reinforce that additional efforts to provide vaccination services in communities play an important role in reaching families who do not interact with the fixed health facility. When considering opportunities to integrate maternal, child health, and immunization services, it is important to consider the potential heterogeneity among mothers with poor access to care and how this may impact care-seeking for themselves and their children.

While data limitations prevented us from looking specifically at remote rural and poor urban areas–settings identified as areas with a high proportion or number of zero dose children–we conducted an analysis to assess the distribution of household wealth across urban and rural areas [10]. In the full population, 1 in 5 children (20.8%) are in the poorest wealth quintile and live in rural areas, but among zero dose children, this proportion is higher; more than 1 in 3 zero dose children (35.0%) are in the poorest wealth quintile and live in rural areas [45]. This is similar in low and lower-middle income countries and shows that children in poorer households in rural areas make up a substantial proportion of the zero dose population, and contribute more to the zero dose burden than would be expected on the basis of the distribution of households by wealth and urban/rural residence alone. Although in our study, the population of children in urban areas is a little less than half the size of those living in rural areas (34.7% vs. 65.3%, respectively), there are 3.5 times as many children in the wealthiest quintile in urban areas than in rural areas (12.8% vs. 3.7%, respectively). Thus, the overall urban population in our sample is considered wealthier than the rural population. This partially explains why the urban zero dose population skews, paradoxically, towards the wealthier quintiles. Additional data and methods are needed to have a more nuanced understanding of the relationship between household wealth and zero dose status in children in urban areas, especially given that in some contexts, the poorest urban children fare worse than their rural peers [46].

By assessing the zero dose status in children 12–23 months of age, we can assess recent performance of routine infant immunizations. In many contexts, immunization programs in the second year of life were still being introduced and strengthened over the past decade, so limiting our analysis to vaccines received in the first year of life provides a more precise and consistent overview of zero dose status across the countries [38]. Although a single-vaccine definition has also been used to define zero dose status (i.e., no receipt of DTP/pentavalent vaccine), our multi-vaccine definition of zero dose status provides a conservative estimate of the number of zero dose children and aims to identify those who truly have not received any vaccines through routine services, campaigns, or other outreach activities. A forthcoming analysis aims to understand how using a “no DTP 1” definition of zero dose status compares with using a “purist” definition of having not received any vaccine doses, as zero dose. The analysis will examine quantitative and qualitative implications of the two definitions on describing the population of zero dose children.

A strength of our analysis includes utilizing recent data from 82 LMICs. We utilized a theory-driven approach to guide our model selection by developing and using a robust, multi-level conceptual framework. Due to the design of DHS and MICS that utilized multi-level complex sampling, our results are nationally representative, and we have large power to make statistical inferences. In addition, stratification by country income group provides insights to characterize zero dose status and risk factors. Selecting DHS/MICS datasets for our analysis enabled us to utilize recent data on a set of household and child health indicators that could be compared and analyzed across 82 countries. Yet, a limitation is that there may be correlates of zero dose status that are not collected in DHS/MICS questionnaires. From recent work, we know that certain settings (remote rural, urban poor, and conflict-affected settings) are home to a disproportionate number of zero dose children, but we were not able to factor this into our analysis due to data availability in the DHS/MICS [10]. Primarily, poor urban and peri-urban communities are reported to be under-sampled in large national surveys such as the DHS [47,48]. Households in poor urban communities are challenging to enumerate, and census data, which is used to enumerate areas in the DHS and MICS, often miss vulnerable populations present in urban areas, such as migrants and homeless families [47,48]. Access to vulnerable urban households and communities is often limited and survey data collection is therefore especially difficult in these settings, which results in the under-representation of urban poor populations in surveys such as the DHS and MICS [47,48]. In addition, DHS and MICS use an asset-based indicator to assess household-level wealth, which has limitations in describing urban and rural population socio-economic status in LMICs [49,50]. The asset index does not consider whether an asset is new or old, and lacks a denominator that adjusts for the cost of living across settings, so therefore misses out on important comparisons (i.e., ownership of certain assets may be more common in rural vs. urban areas) [51]. Hence, this limits the ability of our analysis to provide a close examination between urban poverty and zero dose status. Although we acknowledged these limitations, we used the standard wealth quintile index to allow for comparability with other studies, as this relatively straightforward index is easily understood and widely utilized [52]. Several indices to measure poverty, equity, and household wealth have been proposed and should be explored in future analyses that examine the relationship between urban living, household economic status, and zero dose status.

There is somewhat limited interpretability of our analysis at the global level and among upper-middle income countries, as we do not include high income countries and many upper-middle income countries in our analysis. Although higher income countries are not expected to account for a large number of zero dose children, our findings may not be applicable in these settings, and understanding the children yet to be reached by any immunization services in contexts with potentially stronger immunization access and coverage may be important to developing solutions in these contexts [53]. Acknowledging that the countries in our analysis constitute approximately two-thirds of the global birth cohort, our findings are interpretable at the global level, country income group level, and national level, but we do not consider sub-national disparities. We expect within-country disparities to occur and to be relevant to zero dose communities, potentially more so in upper-middle income countries, where there may be greater inequities between zero dose children and vaccinated children [54]. In addition, our data is from 2011 to 2020, with 2017 as the median survey year, so these findings may not show the current setting, especially as health system resources and capacity may have shifted due to the COVID-19 pandemic. Lastly, we had fewer surveys from upper-middle income countries, since DHS/MICS have not conducted recent surveys in these countries, and we included fewer upper-middle income countries in our multivariate analysis due to missing data. Our sample size of zero dose children in upper-middle income countries was comparatively more limited, so some of our findings in upper-middle countries were not statistically significant and should be interpreted with some caution. Further analyses should incorporate alternative data sources and qualitative data to more fully understand the zero dose population and risk factors in upper-middle income countries.

Future work should focus on identifying best practices and approaches to identify and reach unimmunized children. Although there is extensive literature on improving access to health services and continued engagement with the health system, it is becoming clear that zero dose children experience unique barriers that can exclude them from the health system entirely; it is therefore necessary to identify, develop, and scale the types of interventions–such as improved community-based microplanning–that can respond to these barriers and engage them with the immunization system for the first time. In addition, since they have limited interaction with the health system, reaching zero dose children with vaccinations could be an opportunity to integrate other health services and strengthen overall maternal and child health programs, and vice versa. Innovative strategies to improve integration of services while strengthening existing immunization platforms, such as birth dose immunization, should be explored to better protect the health of zero dose children. This study also underscores the association between access to maternal health services and immunization uptake and makes an argument for stronger collaboration between maternal and newborn health and immunization managers.

As presented in our framework, contextual factors, health systems factors, and individual psychosocial factors are also drivers of zero dose status, so future work should aim to understand and quantify the contribution of these areas to the burden of zero dose status. It is promising to see global commitment focused on improving immunization equity and on reaching the most vulnerable children. It is important that in filling these research gaps, context-specific approaches are identified and implemented to address the multi-level barriers faced by zero dose children.

## Conclusions

Across the 82 low- and middle-income countries in our analysis, 7.5% of children 12 to 23 months have not received a single dose of BCG, polio, pentavalent, and measles-containing vaccines and are considered ‘zero dose’. Half (51.9%) of zero dose children live in African countries, half (51.5%) have mothers who have not received any formal education, and 37.5% live in the poorest wealth quintile. Limited to no access to maternal health care was a strong risk factor for zero dose status, with children of mothers without ANC visits and tetanus injections and who had home deliveries significantly more likely to be zero dose than their peers with mothers who did access these services, when controlled for other factors. Yet, the relationship between maternal care and zero dose status highlights an important paradox: while zero dose children are likely to have mothers with limited access to care, a substantial proportion of zero dose children have mothers who have repeatedly accessed care. This underscores important opportunities to strengthen the quality of maternal care and improve the integration of maternal, neonatal, and child health programs to mitigate missed opportunities for vaccination as an approach to reach zero dose children.

## Supporting information

S1 Appendix National prevalence of zero dose status, per most recent DHS/MICS conducted between 2011 and 2020. (DOCX)

S2 Appendix Distribution of demographic characteristics among children 12–23 months, globally and by country income level. (DOCX)

S3 Appendix Distribution of demographic characteristics and potential risk factors among children 12–23 months, stratified by whether countries were included or excluded in our multivariate analysis. (DOCX)
