# Supplementary material for: Understanding household-level risk factors for zero dose immunization in 82 low- and middle-income countries
Source: PLoS One. 2023 Dec 7;18(12):e0287459. doi: 10.1371/journal.pone.0287459 (PMC10703331; doi:10.1371/journal.pone.0287459)
Supplement: S1 Appendix — (DOCX) [file pone.0287459.s001.docx]

**APPENDIX**

**S1 Appendix 1**: **National prevalence of zero dose status, per most recent DHS/MICS conducted between 2011 and 2020**

| **Country** | **Year of survey** | **Zero dose prevalence (95% CI)** | **N** |
| --- | --- | --- | --- |
| Afghanistan | 2015 | 13.07 (11.21, 15.17) | 5,820 |
| Albania | 2018 | 8.80 (5.65, 13.47) | 543 |
| Algeria | 2019 | 2.15 (1.56, 2.95) | 2,953 |
| Angola | 2016 | 19.97 (17.44, 22.77) | 2,842 |
| Armenia | 2016 | 0.83 (0.33, 2.06) | 350 |
| Bangladesh | 2018 | 1.39 (0.81, 2.37) | 1,656 |
| Belize | 2016 | 1.64 (0.54, 4.91) | 503 |
| Benin | 2018 | 10.94 (9.21, 12.95) | 2,521 |
| Burkina Faso | 2010 | 1.82 (1.21, 2.73) | 2,791 |
| Burundi | 2017 | 0.32 (0.15, 0.68) | 2,645 |
| Cambodia | 2014 | 2.42 (1.63, 3.58) | 1,441 |
| Cameroon | 2018 | 9.98 (8.20, 12.08) | 1,821 |
| Central African Republic | 2019 | 28.92 (25.57, 32.52) | 1,688 |
| Chad | 2015 | 18.52 (16.12, 21.20) | 2,880 |
| Comoros | 2012 | 10.67 (8.20, 13.78) | 630 |
| Congo | 2015 | 6.29 (4.91, 8.04) | 1,773 |
| Costa Rica | 2018 | 0.12 (0.03, 0.57) | 708 |
| Côte d Ivoire | 2016 | 1.73 (1.14, 2.62) | 1,784 |
| Cuba | 2019 | 0.89 (0.22, 3.53) | 1,119 |
| DRC | 2018 | 19.93 (17.19, 22.98) | 4,251 |
| Egypt | 2014 | 0.03 (0.01, 0.14) | 3,281 |
| El Salvador | 2014 | 0.42 (0.09, 1.94) | 1,504 |
| Ethiopia | 2016 | 15.02 (12.35, 18.16) | 1,953 |
| Gabon | 2012 | 4.83 (3.33, 6.95) | 1,197 |
| Gambia | 2018 | 1.49 (0.95, 2.33) | 1,895 |
| Ghana | 2014 | 1.61 (0.91, 2.85) | 1,128 |
| Guatemala | 2015 | 0.61 (0.35, 1.07) | 2,408 |
| Guinea | 2018 | 22.48 (19.28, 26.04) | 1,422 |
| Guinea-Bissau | 2019 | 5.27 (3.68, 7.49) | 1,409 |
| Guyana | 2014 | 2.83 (1.49, 5.29) | 688 |
| Haiti | 2017 | 10.24 (7.92, 13.13) | 1,206 |
| Honduras | 2011 | 0.45 (0.20, 1.02) | 2,277 |
| India | 2016 | 6.06 (5.72, 6.43) | 49,284 |
| Indonesia | 2017 | 6.96 (5.90, 8.18) | 3,535 |
| Iraq | 2018 | 3.00 (2.34, 3.85) | 3,205 |
| Jordan | 2018 | 6.68 (5.02, 8.85) | 1,958 |
| Kazakhstan | 2015 | 1.22 (0.70, 2.10) | 1,103 |
| Kenya | 2014 | 1.73 (1.19, 2.51) | 4,052 |
| Kiribati | 2019 | 38.25 (33.29, 43.46) | 453 |
| Kyrgyzstan | 2018 | 2.39 (1.21, 4.63) | 643 |
| Lao PDR | 2017 | 13.01 (11.32, 14.91) | 2,215 |
| Lesotho | 2018 | 2.49 (1.36, 4.51) | 667 |
| Liberia | 2013 | 1.66 (1.01, 2.72) | 1,433 |
| Madagascar | 2018 | 18.63 (16.32, 21.17) | 2,590 |
| Malawi | 2016 | 1.68 (1.13, 2.49) | 3,245 |
| Maldives | 2017 | 8.21 (5.71, 11.67) | 587 |
| Mali | 2018 | 14.24 (11.67, 17.27) | 1,961 |
| Mauritania | 2015 | 9.66 (7.74, 11.99) | 2,131 |
| Mexico | 2015 | 5.56 (3.66, 8.38) | 1,536 |
| Mongolia | 2018 | 1.31 (0.71, 2.42) | 1,077 |
| Mozambique | 2011 | 4.88 (3.67, 6.47) | 2,225 |
| Myanmar | 2016 | 7.90 (5.40, 11.40) | 915 |
| Namibia | 2013 | 4.44 (2.88, 6.77) | 991 |
| Nepal | 2019 | 2.74 (1.77, 4.23) | 1,327 |
| Niger | 2012 | 4.39 (3.39, 5.67) | 2,151 |
| Nigeria | 2018 | 19.34 (17.47, 21.36) | 6,084 |
| Pakistan | 2018 | 3.77 (2.63, 5.39) | 2,331 |
| Papua New Guinea | 2018 | 24.42 (20.94, 28.28) | 1,838 |
| Paraguay | 2016 | 2.60 (1.71, 3.93) | 1,012 |
| Peru | 2012 | 1.15 (0.68, 1.92) | 1,861 |
| Philippines | 2017 | 9.74 (7.93, 11.90 | 1,996 |
| Rwanda | 2015 | 0.68 (0.35, 1.33) | 1,537 |
| Sao Tome and Principe | 2019 | 1.16 (0.43, 3.09) | 349 |
| Senegal | 2019 | 3.23 (2.08, 4.99) | 1,178 |
| Serbia | 2019 | 1.69 (0.67, 4.22) | 384 |
| Sierra Leone | 2019 | 2.42 (1.67, 3.49) | 1,864 |
| South Africa | 2016 | 5.77 (3.82, 8.63) | 664 |
| Sudan | 2014 | 12.03 (9.86, 14.60) | 2,641 |
| Suriname | 2018 | 12.60 (9.39, 16.70) | 763 |
| Swaziland | 2014 | 1.62 (0.77, 3.38 | 541 |
| Tajikistan | 2017 | 3.57 (2.62, 4.85) | 1,270 |
| Tanzania | 2016 | 1.99 (1.29, 3.05) | 2,137 |
| Thailand | 2019 | 0.50 (0.27, 0.92) | 2,879 |
| Timor-Leste | 2016 | 19.06 (16.45, 21.98) | 1,443 |
| Togo | 2017 | 4.61 (3.18, 6.65) | 973 |
| Tonga | 2019 | 1.00 (0.32, 3.09) | 246 |
| Tunisia | 2018 | 1.06 (0.51, 2.22) | 656 |
| Uganda | 2016 | 1.43 (0.98, 2.07) | 2,907 |
| Viet Nam | 2014 | 2.34 (1.39, 3.92) | 785 |
| Yemen | 2013 | 16.30 (14.50, 18.27) | 3,053 |
| Zambia | 2018 | 1.40 (0.89, 2.21) | 1,913 |
| Zimbabwe | 2019 | 5.02 (3.47, 7.21) | 1,153 |
