# Supplementary material for: Understanding household-level risk factors for zero dose immunization in 82 low- and middle-income countries
Source: PLoS One. 2023 Dec 7;18(12):e0287459. doi: 10.1371/journal.pone.0287459 (PMC10703331; doi:10.1371/journal.pone.0287459)
Supplement: S2 Appendix — (DOCX) [file pone.0287459.s002.docx]

**S2 Appendix 2: Distribution of demographic characteristics among children 12-23 months, globally and by country income level**

|  | **Global**  N = 194,829  (% of population) | | | **Low-income countries**  N = 43,884  (% of population) | | **Lower-middle income countries**  N = 137,702  (% of population) | | **Upper-middle income countries**  N = 13,243  (% of population) |
| --- | --- | --- | --- | --- | --- | --- | --- | --- |
| **WHO region** |  | | |  | |  | |  |
| AFR | 36.5 | | | 90.5 | | 21.1 | | 17.4 |
| AMR | 4.2 | | | 1.4 | | 1.0 | | 46.9 |
| EMR | 15.1 | | | 6.9 | | 17.1 | | 21.9 |
| EUR | 1.0 | | | 1.2 | | 0.3 | | 7.3 |
| SEAR | 37.8 | | | 0.0 | | 52.8 | | 6.5 |
| WPR | 5.4 | | | 0.0 | | 7.7 | | 0.0 |
| **Rural living** | 65.3 | | | 73.9 | | 65.5 | | 34.6 |
| **Sex of Child, female** | 48.9 | | | 50.1 | | 48.4 | | 50.1 |
| **Wealth Index in Quintiles** | | |  | |  | |  | |
| Richest | 16.5 | | | 16.5 | | 16.8 | | 14.0 |
| Richer | 19.1 | | | 19.3 | | 19.1 | | 18.3 |
| Middle | 20.5 | | | 20.6 | | 20.4 | | 20.5 |
| Poorer | 21.2 | | | 21.2 | | 21.0 | | 23.7 |
| Poorest | 22.8 | | | 22.4 | | 22.8 | | 23.5 |
| **Number of children** |  | | |  | |  | |  |
| 1 | 27.2 | | | 19.5 | | 29.3 | | 30.7 |
| 2-4 | 54.9 | | | 48.0 | | 56.6 | | 59.7 |
| >5 | 17.9 | | | 32.5 | | 14.1 | | 9.6 |
| *Missing (%)* | 0.5 | | | 0.8 | | 0.3 | | 2.0 |
| **Adolescent age of mother (15-19 years)** | 6.1 | | | 8.2 | | 5.2 | | 8.5 |
| *Missing (%)* | 0.5 | | | 0.7 | | 0.2 | | 1.9 |
| **Maternal Education** |  | | |  | |  | |  |
| Primary | 23.2 | | | 35.2 | | 19.6 | | 21.2 |
| None | 27.5 | | | 40.8 | | 25.5 | | 4.7 |
| Secondary or higher | 49.3 | | | 24.0 | | 55.0 | | 74.1 |
| *Missing (%)* | 0.5 | | | 0.7 | | 0.3 | | 1.9 |
| **Access to maternal care** | | |  | |  | |  | |
| **Maternal Tetanus Injection** | |  | |  | |  | |  |
| >2 times | | 62.4 | | 53.7 | | 66.0 | | 53.5 |
| 1 time | | 20.1 | | 23.7 | | 17.7 | | 35.9 |
| 0 times | | 17.5 | | 22.7 | | 16.4 | | 10.6 |
| *Missing (%)* | | 14.6 | | 12.6 | | 14.1 | | 26.5 |
| **Number of Antenatal Visit** | |  | |  | |  | |  |
| >4 visits | | 59.8 | | 47.4 | | 60.9 | | 89.3 |
| 1-3 visits | | 28.6 | | 40.2 | | 26.7 | | 9.1 |
| 0 visits | | 11.6 | | 12.3 | | 12.3 | | 1.6 |
| *Missing (%)* | | 7.2 | | 8.8 | | 6.5 | | 9.6 |
| **Place of Delivery** | |  | |  | |  | |  |
| Medical Facilities | | 72.0 | | 63.0 | | 72.8 | | 94.1 |
| Home | | 27.4 | | 36.0 | | 26.7 | | 5.7 |
| Other | | 0.7 | | 1.1 | | 0.6 | | 0.3 |
| *Missing (%)* | | 0.8 | | 1.2 | | 0.4 | | 3.5 |
| **Access to media** | |  | |  | |  | |  |
| **Frequency of Listening to Radio** | | | |  | |  | |  |
| Almost daily | | 4.0 | | 3.3 | | 2.8 | | 21.9 |
| At least weekly | | 18.0 | | 26.6 | | 14.4 | | 28.3 |
| Less than weekly | | 78.0 | | 70.1 | | 82.8 | | 49.8 |
| *Missing (%)* | | 5.9 | | 1.5 | | 5.5 | | 24.5 |
| **Frequency of Watching TV** | |  | |  | |  | |  |
| Almost daily | | 20.9 | | 3.1 | | 25.5 | | 37.8 |
| At least weekly | | 29.0 | | 16.5 | | 33.0 | | 31.4 |
| Less than weekly | | 50.2 | | 80.4 | | 41.5 | | 30.9 |
| *Missing (%)* | | 6.3 | | 1.5 | | 6.0 | | 24.6 |
