# Supplementary material for: Understanding household-level risk factors for zero dose immunization in 82 low- and middle-income countries
Source: PLoS One. 2023 Dec 7;18(12):e0287459. doi: 10.1371/journal.pone.0287459 (PMC10703331; doi:10.1371/journal.pone.0287459)
Supplement: S3 Appendix — (DOCX) [file pone.0287459.s003.docx]

**S3 Appendix 3**: **Distribution of demographic characteristics and potential risk factors among children 12-23 months, stratified by whether countries were included or excluded in our multivariate analysis**

|  | **Lower middle income countries** | | |  | **Upper middle income countries** | |  |
| --- | --- | --- | --- | --- | --- | --- | --- |
|  | Included  N = 127,526  (% of population) | Excluded  N = 10,176  (% of population) | p-value* | | Included  N = 6,636  (% of population) | Excluded  N = 6,606  (% of population) | p-value* |
| **Zero dose prevalence** | 7.2 | 4.6 | <0.001 | | 3.8 | 4.1 | <0.001 |
| **Rural living** | 65.1 | 71.2 | <0.001 | | 33.6 | 35.6 | 0.500 |
| **Sex of Child, female** | 48.3 | 49.5 | 0.200 | | 47.7 | 52.5 | 0.013 |
| **Wealth Index in Quintiles** |  |  | 0.043 | |  |  | 0.700 |
| Richest | 16.6 | 18.7 |  | | 15.0 | 12.9 |  |
| Richer | 19.0 | 20.2 |  | | 18.2 | 18.3 |  |
| Middle | 20.4 | 19.9 |  | | 20.8 | 20.3 |  |
| Poorer | 21.0 | 21.2 |  | | 23.2 | 24.2 |  |
| Poorest | 23.0 | 20.1 |  | | 22.8 | 24.2 |  |
| **Maternal Education** |  |  | <0.001 | |  |  | <0.001 |
| Secondary or higher | 55.0 | 55.1 |  | | 65.8 | 82.7 |  |
| Primary | 18.8 | 29.0 |  | | 26.7 | 15.6 |  |
| None | 26.2 | 15.9 |  | | 7.4 | 1.8 |  |
| *Missing (%)* | 0.2 | 1.2 |  | | 0.3 | 3.5 |  |
| **Number of children** |  |  | <0.001 | |  |  | <0.001 |
| 1 | 29.3 | 29.3 |  | | 27.7 | 33.8 |  |
| 2-4 | 56.7 | 55.3 |  | | 57.7 | 61.8 |  |
| >5 | 14.0 | 15.3 |  | | 14.6 | 4.4 |  |
| *Missing (%)* | 0.2 | 0.7 |  | | 0.3 | 3.7 |  |
| **Adolescent age of mother (15-19 years)** | 4.6 | 13.0 | <0.001 | | 8.1 | 8.9 | <0.001 |
| *Missing (%)* | 0.2 | 0.6 |  | | 0.2 | 3.5 |  |
| **Marital status** |  |  | <0.001 | |  |  | <0.001 |
| Currently married | 96.2 | 97.9 |  | | 77.0 | 82.4 |  |
| Formerly married | 2.2 | 1.8 |  | | 3.6 | 10.9 |  |
| Never married | 1.6 | 0.3 |  | | 19.4 | 6.7 |  |
| *Missing (%)* | 0.2 | 0.6 |  | | 0.2 | 3.7 |  |
| **Female head of household** | 13.0 | 13.0 | >0.900 | | 24.6 | 25.2 | 0.800 |
| **Access to maternal care** | | | | | | | |
| **Maternal Tetanus Injection** |  |  | <0.001 | |  |  | <0.001 |
| >2 times | 66.2 | 54.3 |  | | 47.4 | 60.6 |  |
| 1 time | 17.2 | 45.6 |  | | 33.0 | 39.4 |  |
| 0 times | 16.6 | 0.1 |  | | 19.6 | 0.0 |  |
| *Missing (%)* | 8.9 | 80.5 |  | | 20.7 | 32.3 |  |
| **Number of Antenatal Visit** |  |  | <0.001 | |  |  | <0.001 |
| >4 visits | 61.4 | 55.3 |  | | 82.4 | 96.5 |  |
| 1-3 visits | 25.8 | 39.5 |  | | 14.7 | 3.4 |  |
| 0 visits | 12.9 | 5.2 |  | | 2.9 | 0.2 |  |
| *Missing (%)* | 6.3 | 9.0 |  | | 8.9 | 10.3 |  |
| **Place of Delivery** |  |  | <0.001 | |  |  | <0.001 |
| Medical Facilities | 74.7 | 47.7 |  | | 90.6 | 97.8 |  |
| Home | 24.7 | 52.0 |  | | 9.1 | 2.0 |  |
| Other | 0.6 | 0.3 |  | | 0.4 | 0.2 |  |
| *Missing (%)* | 0.3 | 1.4 |  | | 0.5 | 6.5 |  |
| **Access to media** | | | | | | | |
| **Frequency of Listening to Radio** |  |  | <0.001 | |  |  | <0.001 |
| Almost daily | 2.9 | 1.0 |  | | 11.7 | 41.1 |  |
| At least weekly | 15.0 | 4.2 |  | | 22.1 | 40.0 |  |
| Less than weekly | 82.1 | 94.8 |  | | 66.2 | 18.9 |  |
| *Missing (%)* | 3.5 | 31.3 |  | | 1.6 | 47.5 |  |
| **Frequency of Watching TV** |  |  | <0.001 | |  |  | <0.001 |
| Almost daily | 26.6 | 5.5 |  | | 44.9 | 24.0 |  |
| At least weekly | 31.8 | 53.0 |  | | 32.3 | 29.5 |  |
| Less than weekly | 41.5 | 41.6 |  | | 22.8 | 46.4 |  |
| *Missing (%)* | 4.0 | 31.0 |  | | 1.1 | 48.3 |  |

**Note**: Only one low-income country was excluded from the multivariate analysis, accounting for 1.2% of our sample size in low-income countries, so comparative descriptive analyses were included only for lower-middle and upper-middle income countries.

* difference in proportions between zero dose and non-zero dose children, p<0.05
